# Accurate Classification of RNA Structures Using Topological Fingerprints

**DOI:** 10.1371/journal.pone.0164726

**Published:** 2016-10-18

**Authors:** Jiajie Huang, Kejie Li, Michael Gribskov

**Affiliations:** 1 Department of Biological Sciences, Purdue University, West Lafayette, Indiana, United States of America; 2 Life Sciences Solutions Group, Thermo Fisher Scientific, South San Francisco, California, United States of America; 3 Computational Biology Department, Biogen Idec, Cambridge, Massachusetts, United States of America; 4 Department of Computer Science, Purdue University, West Lafayette, Indiana, United States of America; Ben-Gurion University, ISRAEL

## Abstract

While RNAs are well known to possess complex structures, functionally similar RNAs often have little sequence similarity. While the exact size and spacing of base-paired regions vary, functionally similar RNAs have pronounced similarity in the arrangement, or topology, of base-paired stems. Furthermore, predicted RNA structures often lack pseudoknots (a crucial aspect of biological activity), and are only partially correct, or incomplete. A topological approach addresses all of these difficulties. In this work we describe each RNA structure as a graph that can be converted to a topological spectrum (RNA fingerprint). The set of subgraphs in an RNA structure, its RNA fingerprint, can be compared with the fingerprints of other RNA structures to identify and correctly classify functionally related RNAs. Topologically similar RNAs can be identified even when a large fraction, up to 30%, of the stems are omitted, indicating that highly accurate structures are not necessary. We investigate the performance of the RNA fingerprint approach on a set of eight highly curated RNA families, with diverse sizes and functions, containing pseudoknots, and with little sequence similarity–an especially difficult test set. In spite of the difficult test set, the RNA fingerprint approach is very successful (ROC AUC > 0.95). Due to the inclusion of pseudoknots, the RNA fingerprint approach both covers a wider range of possible structures than methods based only on secondary structure, and its tolerance for incomplete structures suggests that it can be applied even to predicted structures. Source code is freely available at https://github.rcac.purdue.edu/mgribsko/XIOS_RNA_fingerprint.

## Introduction

Once seen as a simple scaffold, RNA is now known to play important regulatory and catalytic roles. RNA is involved in processes including transcriptional regulation [1], RNA maturation and modification [2], and RNA splicing [3]. The structural motifs in RNA that are responsible for its functions are evolutionarily conserved; however, unlike DNA and protein, for which conserved functional motifs can be identified based on sequence similarity, the functional motifs in RNA may have little or no sequence similarity [4], and instead conserve patterns of base-pairing (stems) and topological relationships between base-paired regions, for instance nesting of stems, multi-loops, and pseudoknots [5, 6]. This topological view of RNA structure has been discussed by Giegerich *et al*. who point out that, in a family of RNAs with the same function, the global arrangements of structural elements (topology) are conserved, but there is considerable variation in the length of stems, presence of bulge loops and unpaired bases, and type of base-pairs. Therefore, in the study of RNA functions, it may be more relevant to look at global topological patterns than individual base-pairs [7, 8]. RNAs with similar functions, for example those in ribonuclease P (RNase P), the ribosome, or self-splicing introns, typically have strongly conserved topologies [5, 9–11]. One of the notable topological aspects of RNA structure is the importance of pseudoknots in many classes of molecules. For example, in Hepatitis Delta Virus (HDV), a double-pseudoknotted structure contained in a self-cleaving ribozyme is a key factor in HDV infection [12]; in Group I self-splicing introns, the catalytic core is formed by pseudoknots [13]; in ribosomal RNA, pseudoknots at the catalytic site are the key structures that mediate microbial resistance to antibiotics [14] and stimulate viral frame-shifting [15].

As only a small number of functional RNA classes have been identified, we believe that the majority of regulatory and functional RNA motifs are yet to be identified. Eukaryotic genomes are pervasively transcribed [16]; almost every base can be found in an RNA transcript. This is surprising since, in most genomes, protein-coding sequences comprise only a small fraction of the genome. Much of this RNA is therefore likely to be regulatory in nature, and will almost certainly contain functionally important structures, including pseudoknots.

Just as conserved structural topologies are important for RNA function, the identification of novel conserved topologies provides an approach to discovering the functions of currently unknown classes of biologically important RNAs. An analogy can be made to the importance of sequence alignment and database searching programs in identifying novel proteins and DNA regulatory elements. While typical functional RNA structures are pseudoknotted, the current computational approaches to RNA structure comparison only consider structures without pseudoknots. Because of their importance to RNA function, we believe that incorporating pseudoknots in structural comparisons is critical to identifying biologically important classes of molecules. In this paper we propose a straightforward approach to comparing RNA structural topologies, including pseudoknots, and identifying known and unknown conserved topologies. Waterman [17] introduced the first graphical representation of RNA structure, the full-graph, where the nodes represents nucleotides. The tree-graph representation was later introduced by Shapiro *et al*., which is an abstract tree where the nodes represent structural elements [18–20], and this coarse-grained representation was implemented in the ViennaRNA package [21]. Fontana *et al*. implemented the homeomorphically irreducible tree (HIT) that represents an RNA secondary structure as a contracted topology in which each node represents a structural element weighted by size [22]. Shu *et al*. have developed the element-contact graphs (ECGs) with size-weighted nodes as well [23], which uses topological indices, such as the Randić index [23, 24], the Wiener index, and Balaban index, to measure graph connectivity [23, 24]. Although the ECGs framework and an extended Wiener index [25] were developed to be able to identify small ncRNAs such as miRNAs, no evidence was shown for its ability to classify larger RNAs (for example, 23S rRNA are usually over 1000nt long) with low sequence similarity. The RNAshapes package [7, 8] of Giegerich *et al*., which represents RNA structures as abstract shapes and aims for efficient RNA structure comparisons, has been shown useful in topologically clustering RNA families; however, RNAshapes does not perform well on families with pseudoknots [26]. Building on this work, Heyne *et al*. developed a graph-based pipeline called GraphClust [27] for fast clustering of RNA molecules. In this approach, RNA secondary structures are generated by the RNAshapes package from input sequences, encoded by graphs preserving nucleotide connectivity, and clustered by a graph kernel, the Neighborhood Subgraph Pairwise Distance Kernel (NSPDK) [28]. However, given data sets of small RNA sequences (sequence length < 400nt, similarity up to 80%) the precision and recall of GraphClust only reaches around 85%. In addition, these approaches do not include pseudoknots in either the representation or the analysis.

The Schlick group has developed the RNA-As-Graphs procedure, which combines elements from several sources to develop a database of mathematically possible RNA graphs in which RNA structures are represented as Shapiro tree graphs, without pseudoknots, or as dual graphs, with inclusion of pseudoknots [29–32]. Numerical descriptors have been applied to comparison of these RNA topological patterns. The eigenvalue spectrum of the Laplacian matrix measures graph compactness and connectivity; λ_2_, the second eigenvalue of the Laplacian matrix [33–35], measures RNA graph similarity. The Schlick group used several structural invariants, including λ_2_ and linear combinations of α and β (the intercept and slope of the eigenvalues of the Laplacian matrix), for categorizing the structural similarity of RNA graphs, and for predicting whether randomly generated RNA topologies are similar to biological examples (RNA-like). These numerical descriptors, however, have never been shown to be able to group RNAs into structural/functional classes. Moreover, these approaches, which rely on a small number of numeric descriptors, cannot identify similarity between specific substructures nested within fairly large graphs (for instance graphs of the size of RNase P RNA, which may have up to 20 vertices).

There are several aspects of RNA structure that make it particularly hard to identify topologically similar structures. Structures from the same functional family may have little or no sequence similarity; they typically have a similar arrangement of stems (topology), but different local base-pairing; our knowledge of the structures may be incomplete due to lack of a high-quality three-dimensional structure or structural prediction; structures may lack biologically important pseudoknots since tractable computational approaches based on dynamic programming often do not include these important features; or in the case of graph comparison, the computation itself may require infeasible amounts of time. The RNA XIOS graph [36] explicitly represents serial, nested, pseudoknotted, and mutually exclusive stems, but finding topologically similar RNA structures requires identifying isomorphous subgraphs common to one or more structures. The coarse-grained approach we describe here builds on the XIOS approach, addresses the problems described above, and provides a feasible approach to clustering and identifying biological RNAs with topologically similar structures. A coarse-grained RNA secondary structure representation has also been used to predict RNA deleterious mutations by RNAMute [37] and RNAmutants [38]. In addition, a coarse-grained approach is also applied for RNA design such as RNAexinv [39] and Nanofolder [40]. We demonstrate the utility of the XIOS approach by classifying a representative set of pseudoknot-including RNA structural families that have very low levels of sequence similarity–the high accuracy of the classification indicates that this approach can be broadly applied to identifying RNAs with conserved topologies, whether their function is known or unknown.

## Materials and Methods

### Curated RNA families

A set of curated RNA structures have been collected from the literature and a variety of biological databases [41], and is extended in this work (S1 Table). This set of known structures has been carefully selected to contain pseudoknots, to cover a broad range of lengths, and to have been the subject of extensive expert curation by the biological community. This curated set includes 206 structures of transfer RNA, Ribonuclease P RNA, transfer-messenger RNA, group I and group II self-splicing introns, and 5S, 16S and 23S ribosomal RNA. The structures in this curated set have been reviewed to ensure they reflect expert opinion on the correct structure, and to ensure that the reported structures are as accurate as possible given existing experimental data such as X-ray crystallography [42, 43] and covariance analysis [44]. The curated structures have been screened to ensure that all structures are full-length, and no pair of structures has greater than 50% sequence identity. Multiple families of the curated structures contain pseudoknots. While several large databases of RNA structures exist, for instance Rfam [45] and RNAStrand [46], these resources could not be directly used for testing in this work because many families lack pseudoknots, lack a consensus of expert opinion on the correct structure(s), have only a family consensus structure (rather than individual structures for each RNA), have high levels of sequence identity, or comprise incomplete structures or structures in which single stranded regions (or other regions judged to be unimportant) have been removed.

### XIOS graphs

**Fig 1:**
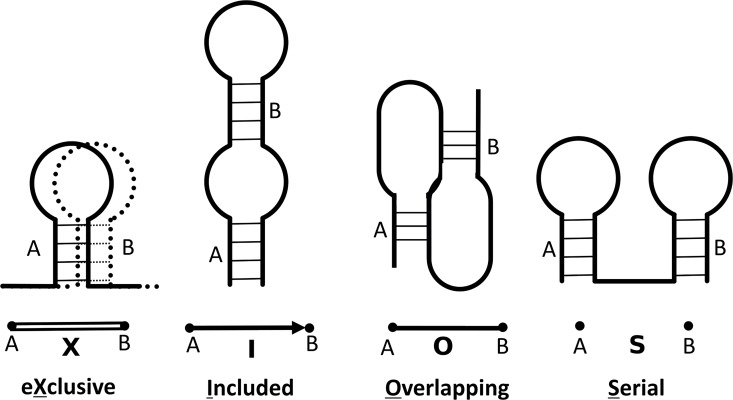
XIOS graph stem-stem relationships. Edges show the relationship between two stems, and may be one of four types: X (mutually exclusive), I (included or nested), O (overlapping or pseudoknotted), or S (serial or adjacent).

In a XIOS graph, RNA stems are shown as vertices and the relationships between stems are shown as edges [36]. Edges may be one of four types: X–mutually exclusive (stems with base conflicts, such as those in two alternative structures that use the same RNA sequence); I–included (nested); O–overlapping (pseudoknotted); S–serial (adjacent) (Fig 1). Because there are exactly four classes, and each pair of stems can have one and only one type of relationship, we can omit S relationships without loss of generality (any pair of vertices without an edge have an implicit S edge). In this work, none of the structures have X edges; the graphs therefore have only two edge types, I and O. S1 Fig shows the XIOS graph representation of the Hepatitis D Virus (HDV) ribozyme RNA.

### Curated XIOS graphs

S1 Table shows the vertex number, edge number, and average degree of the XIOS graphs of the curated RNA structures. Graph matching is highly dependent on the size of the graph (described by the number of vertices and edges) and the average degree of the vertices in the graph; the characteristics of the curated RNA structures differ significantly between families making this a representative set for RNAs in general.

## Results

This work focuses on the topological similarity between RNA structures, that is, similarity in the relative location and nesting of stems, and the location of pseudoknots. In principle, this should provide the broadest range of matching since individual structures often differ in the length of stems and the length of single-stranded regions between stems. As mentioned before, the sequences themselves can be even more variable with little or no sequence conservation detectable, even between RNAs with similar structures. Topologically similar substructures in a pair of RNAs correspond to isomorphous subgraphs in their respective XIOS graphs. The maximal common subgraph (MCS) represents the greatest possible topological match between RNAs, similar to the maximal alignment between two sequences. But the MCS is difficult to identify because of the large size of biologically important structures; *e*.*g*., the 23S rRNA can have more than 50 stems [47]. Finding the MCS of a set of graphs, corresponding to the largest conserved topological motif in a group of RNA structures, is an NP-hard problem [48], making the computational identification of the MCS time consuming. In order to decrease the inefficient scaling inherent in graph matching, we characterize each graph as a set of smaller subgraphs. We call this set of subgraphs the RNA topological fingerprint, or more simply, the RNA fingerprint. There are two key elements needed to determine an RNA fingerprint: a comprehensive dictionary of RNA topological motifs, and an approach to identifying the motifs that are present in a XIOS graph.

### Enumerating a comprehensive set of RNA topologies

We have exhaustively enumerated a non-redundant set of all physically possible RNA topological motifs containing from one to seven stems (Table 1). The graphs in this set are all IO-connected, that is, all vertices (stems) can be reached by traversing I and O edges. Briefly, a complete set of topologies for an N-stem RNA structure can be created by generating all the permutations of an ordered set of 2N numbers; the numbers represent N objects (stems), numbered 1 to N, each with two instances (corresponding to the two base-paired halves of the stem). For three stems (N = 3), the ordered unpermuted set would be (1, 1, 2, 2, 3, 3), with each pair of matching numbers representing the two base-paired halves of a stem. The unpermuted set, above, would thus correspond to three serial stems, and a permuted set such as (1, 2, 3, 2, 3, 1) would indicate a pair of pseudoknotted stems, 2 and 3, found within the loop of stem 1.

**Table 1:** Topological Motif Library.

Number of Stems	Unique Topologies
1	1
2	2
3	8
4	46
5	368
6	3914
7	51390

Obviously, this procedure generates multiple copies (isomorphs) of some topologies, for instance (1, 2, 2, 3, 3, 1) and (3, 1, 1, 2, 2, 3), as well as some graphs that are not connected (for instance the unpermuted set, above). Some of the isomorphs can be eliminated by imposing two restrictions. First, the graph must be connected, and second, the first instances (left half stem) of each object (stem) must occur in numerical order. Even these restrictions do not entirely eliminate permutations that correspond to isomorphic XIOS graphs. For instance, the sets (1, 2, 1, 3, 3, 2) and (1, 2, 2, 3, 1, 3) are mirror images of each other, and correspond to the same XIOS graph. These symmetry-related topologies are detected and removed using the gSpan [36, 49] approach. In gSpan, a graph is described using a canonical labeling called the minimum DFS code; Isomorphic graphs are guaranteed to have identical minimum DFS codes.

Using this approach, we have enumerated a library of all unique physically possible RNA topologies with 2 to 7 stem structures (Table 1). Because the minimum DFS code provides a unique description for each topology, we index the motif library with a compressed version of the minimum DFS code. The index of any structure within the library can be easily determined by simply determining its minimum DFS code.

**Fig 2:**
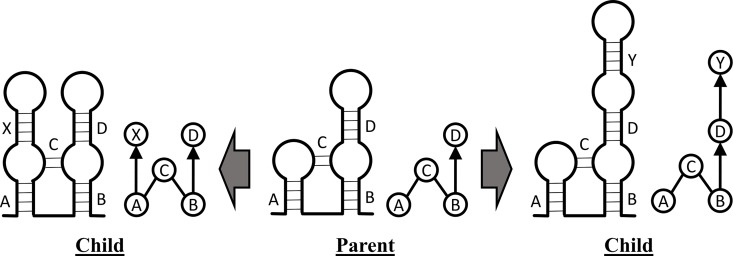
Parent-Child relationships. The parent graph is a 4-stem motif; two different child graphs are created by adding one stem to the parent graph.

The topologies in the library are not independent; two unique 5-stem XIOS graphs, for instance, may share a common 4-stem subgraph as shown in Fig 2. In this situation, we say that the 4-stem subgraph is the parent of both 5-stem graphs because they each have had one stem added to the parent subgraph (Fig 2). When comparing topological motifs, subgraphs that share a parent are clearly more similar than subgraphs that only share a grandparent or great-grandparent. The topological motif library includes all the parent and child relationships between the enumerated graphs in order to allow for partial matching.

### Determining RNA fingerprints using random sampling

**Fig 3:**
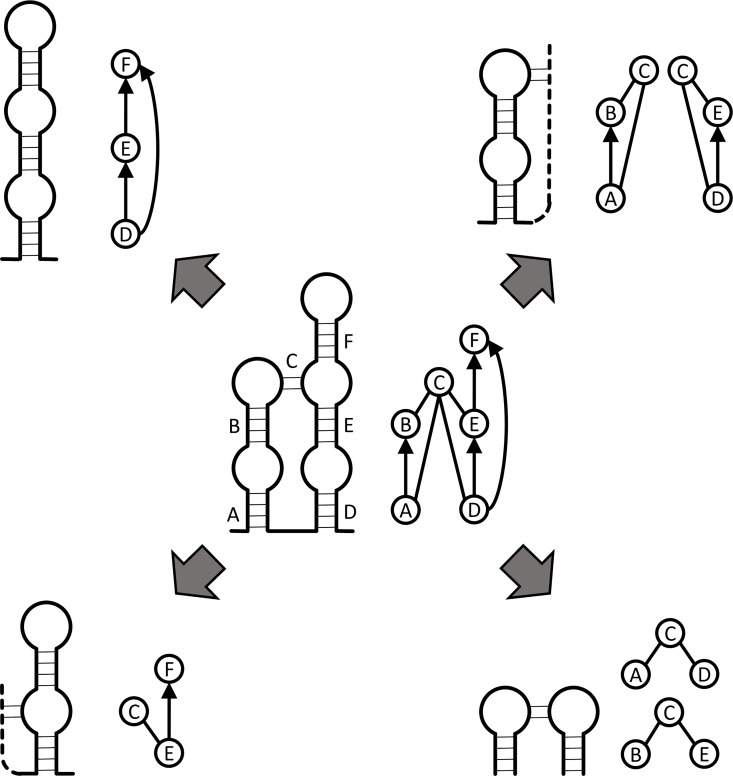
Example of an RNA fingerprint. All 3-vertex motifs (corners) in a 6-vertex RNA graph (center) are shown. The thick solid lines represent RNA chain, the thin solid lines represent base pairs, and the dotted lines represent RNA sequences whose connectivity is not completely specified.

**Fig 4:**
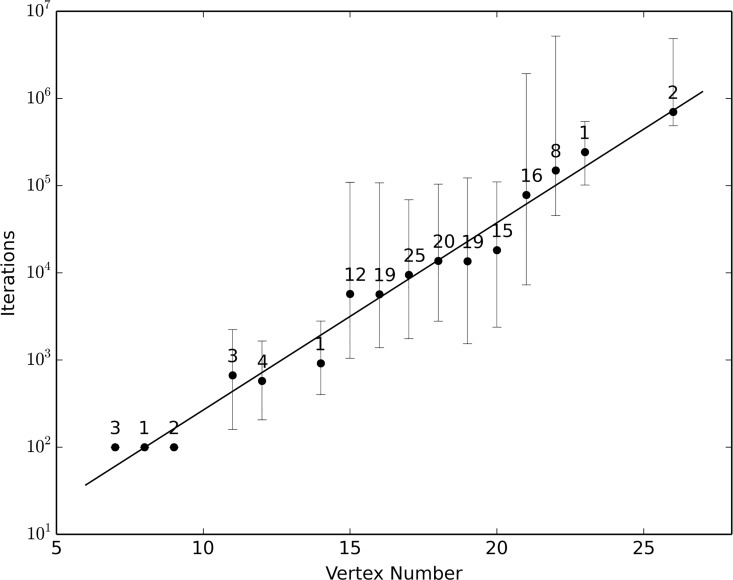
Scaling of sampling with graph size. Fingerprints for 151 RNA graphs in the curated set were determined multiple times (10 times per RNA graph) by random sampling. Numbers above the dots indicate the number of different graphs with the same size (vertex number); each dot represents the average number of iterations needed to determine the complete fingerprint for this specific size group, with bars showing the maximum and minimum iterations as well.

A XIOS graph corresponding to a single structure can be characterized by the set of fixed-size subgraphs it contains. This set of constituent subgraphs is the RNA fingerprint (Fig 3), which can be thought of as a subgraph spectrum that is characteristic of a specific topology. Currently we use a library comprising all 7-stem and smaller subgraphs; this number has been chosen to cover both large and small biological structures, without requiring excessive computation. For even a relatively small graph, for instance a graph with 25 to 30 vertices, exhaustively enumerating the complete set of 7-vertex subgraphs within it can be time consuming. The subgraph sampling approach we describe here allows the determination of the fingerprint in reasonable time on parallel hardware. Briefly, given a XIOS graph, we randomly sample a fixed number, currently seven, of connected vertices from the graph (Table 2). Sampling continues until a suitable termination condition is met, typically when all observed subgraphs have been independently sampled 10 times. In each iteration, one subgraph is sampled and uniquely identified by its minimum DFS code, which is used as a reference to identify the subgraph in the RNA structural motif library. The complete fingerprints of 151 RNA structures computed by an exhaustive method (not shown) have been used to validate the correctness of the RNA fingerprints computed by random sampling (Fig 4).

**Table 2:** Subgraph random sampling pseudocode.

Algorithm: Subgraph Random Sampling
Input: Query graph *G = (V*, *E)*, subgraph size *n*
Output: Sampled subgraph *S = (V*_*s*_, *E*_*s*_*)*
Select a random vertex v_*i*_ *∈ V*
Initialize the set of vertices *V*_*s*_ *= {v*_*i*_*}*
Initialize the set of edges *E*_*s*_ *= {}*
WHILE *| V*_*s*_ *| < n* DO
Identify *N*_*Vs*_, the vertices adjacent to *V*_*s*_
IF *N*_*Vs*_ *= = {}* DO
BREAK
ELSE DO
Select a random vertex *v*_*j*_ from *N*_*Vs*_
Update *E*_*s*_ *= E*_*s*_ *∪ { (v*_*i*_, *v*_*j*_*) } ∀ v*_*i*_, *v*_*j*_ *∈ V*_*s*_
Update *V*_*s*_ *= V*_*s*_ *∪ {v*_*j*_*}*
END IF
END WHILE
RETURN subgraph *S = (V*_*s*_, *E*_*s*_*)*

### RNA fingerprints identify topologically similar RNA structures

The set of subgraph motifs sampled in a query graph is its ***simple fingerprint***. We define the ***extended fingerprint*** as the simple fingerprint plus all of the ancestral subgraphs (i.e., parent, grandparent, etc., see Fig 2) of the simple fingerprint motifs. In this section we use both the simple fingerprint and the extended fingerprint to identify RNAs with similar topologies. The average numbers of motifs in simple and extended fingerprints are shown in S2 Fig.

Consider the simple or extended fingerprints, *X* and *Y*, of RNA *R*_*X*_ and RNA *R*_*Y*_; X = {*x*_*1*_, *x*_*2*_, *x*_*3*_,*…*, *x*_*m*_} and Y = {*y*_*1*_,_,_*y*_*2*_, *y*_*3*_,*…*, *y*_*n*_} where *x*_*1*_, *x*_*2*_, *x*_*3*_, *…*, *x*_*m*_ and *y*_*1*_,_,_*y*_*2*_, *y*_*3*_, *…*, *y*_*n*_ are the subgraph motifs found in RNAs *R*_*X*_ and *R*_*Y*_. We have evaluated five similarity functions (Table 3) for their ability to identify topologically similar structures.

**Table 3:** RNA fingerprint similarity functions. X and Y are fingerprints of the two structures being compared.

Similarity Function	Definition
Intersection	**S_B_**(**X**,**Y**) = |**X** ∩ **Y**|
Cosine [50]	$S_{C}(X,Y)=\frac{\left|X\cap Y\right|}{\sqrt{\left|X||Y\right|}}$
Dice [51, 52]	$S_{D}(X,Y)=\frac{2|X\cap Y|}{\left|X|+|Y\right|}$
Hamming [53]	**S_H_**(**X**,**Y**) = |(**X**⋂**Y**) ⋃ (**X**⋃**Y**)^**C**^|
Jaccard [54]	$S_{J}(X,Y)=\frac{\left|X\cap Y\right|}{\left|X\cup Y\right|}$

**Fig 5:**
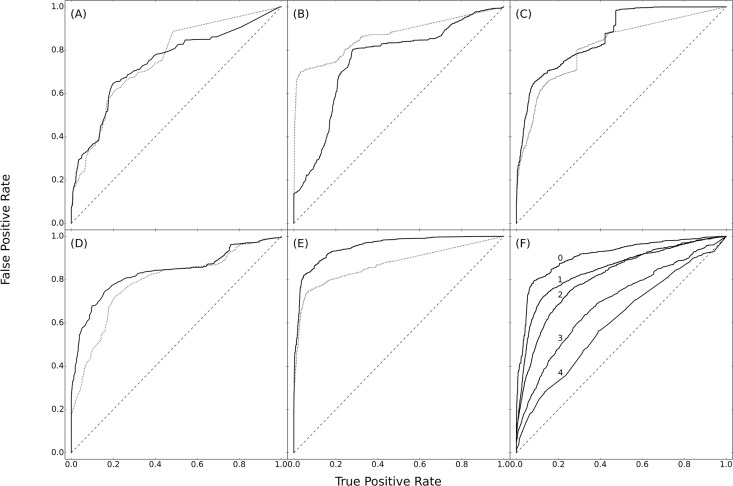
Classification performance of similarity functions. Pairwise similarities were calculated, using the indicated similarity functions, for all RNAs in the curated dataset and ranked from high to low. A pair of RNAs from the same curated family is considered a positive match; otherwise they are considered to be a negative match. In all panels, the dashed line indicates the simple fingerprint, and the solid line the extended fingerprint. The AUC for the simple and extended fingerprints, respectively, are indicated in parentheses, below. (A) Intersection Similarity (AUC simple, 0.759; extended, 0.746), (B) Cosine Similarity (0.867; 0.753), (C) Dice Similarity (0.821; 0.864), (D) Hamming Similarity (0.789; 0.834), and (E) Jaccard Similarity (0.870; 0.952). (F) Classification after random removal of vertices from RNA graphs. All RNAs (except for tRNA and 5S rRNA which are too small for 70% stem removal) are included. The five lines show ROC curves with differing fractions of stems removed (AUC in parentheses): (0) no stem removal (AUC = 0.909), (1) 10% stem removal (0.844), (2) 30% stem removal (0.810), (3) 50% stem removal (0.691), and (4) 70% stem removal (0.605).

**Fig 6:**
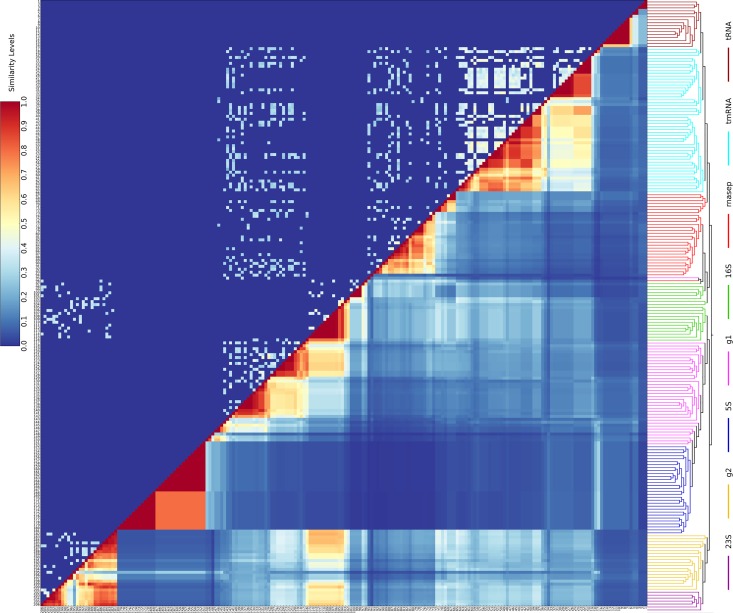
Extended-fingerprint Jaccard similarity between biological RNAs. Upper triangle. Sequence identity. Lower triangle. Extended-fingerprint Jaccard Similarity of all the curated RNA structures (see S3 Fig and S5 Table for IDs). Sequence identity is shown in color, ranging from 0 (blue) to 1 (red) at steps of 0.1. A neighbor-joining dendrogram calculated according to the extended-fingerprint Jaccard similarity is shown on the right side of the heat map.

Fig 5 shows the classification performance of the different similarity functions as measured by Receiver Operating Characteristic (ROC) curves [55]. Jaccard Similarity works best in the classification of RNA structures, with an area under the ROC curve (AUC) greater than 0.95 for the extended fingerprint. The increase in AUC from 0.870 for the simple fingerprint to 0.952 for the extended fingerprint using Jaccard Similarity indicates that the inclusion of parent subgraphs substantially improves the detection of topologically similar structures. The classification performance of Jaccard Similarity using the extended fingerprint on different RNA classes is around 0.95 for all groups except for 16S rRNA and group II introns (S2 Table). Fig 6 shows the ability of the extended-Jaccard similarity to effectively classify the test structures into functional groups. As can be seen in the upper triangle of Fig 6, the level of sequence similarity is very low between these structures and would be insufficient for correct clustering (not shown). The 23S rRNAs form a single group, and also share some similarity with 16S rRNAs, which may be explained by the topological similarity of the two subunits of rRNA [47]. The 5S rRNAs form two separate groups, one with archaeal and eukaryotic nuclear structures, and the other with bacterial structures. Self-splicing introns, especially the Group II Introns, share a high topological similarity with the 23S and 16S rRNAs. The accuracy of the classification confirms that our topological approach can identify topologically similar RNAs, and potentially functionally similar RNAs, as well. In addition, a neighbor-joining tree [56] (Fig 6, S3 and S4 Figs), using the extended-Jaccard similarity, correctly groups almost all the curated RNA families into the correct categories, with only one Group I Intron falling onto a branch outside of its curated group (Fig 6, tree on the right side).

### Similarity of incomplete graphs can be detected using RNA fingerprints

In most cases, topological comparisons must be based on predicted structures, because three-dimensional structures or high-quality comparative structures are usually unavailable. Although structures with pseudoknots can be predicted [41, 57–60], such predicted structures will typically be inaccurate or incomplete. It is highly desirable that a similarity function be able to correctly identify similar RNAs, even when their structures are incomplete. To test the effects of graph incompleteness on the extended-fingerprint Jaccard Similarity function, incomplete RNA graphs were generated by randomly removing a percentage (10%, 30%, 50%, 60%, and 70%, respectively) of the vertices (stems) in the curated structures (Fig 5F). The extended-fingerprint Jaccard Similarity can identify similar structures when only 70% of the original stems are present (AUC = 0.810), and performs better than random even when only 30% of the stems remain. In addition, since pseudoknots are important structural motifs in RNAs, for the 160 RNA structures that have pseudoknots, we generated incomplete RNA graphs by first removing all the pseudoknot-forming vertices (stems), and continuing removing random vertices until 30% of vertices were removed. The extended-fingerprint Jaccard Similarity correctly identifies similar structures with pseudoknots removed (AUC = 0.915, data not shown).

### Fingerprint similarity is not an artifact of graph size

The structures within each curated family generally have very similar numbers of stems. Indeed, one can classify the structures into the correct groups using graph size alone (not shown). It is essential, therefore to consider whether the results in Figs 5 and 6 are merely due to the similarity in sizes. In order to test the effect of size, we have created a test data set in which the graphs have been expanded to the same size (number of vertices) by randomly adding additional vertices and edges to the graphs. In order to ensure that these expanded graphs are typical of real biological structures we use a procedure in which we sample substructures from the set of curated structures, and add them to the curated graphs. In order to do this, we created a database (decoy database) of the 2 to 5 stem motifs found in the curated structures, and randomly added these subgraphs to the curated structures according their frequency in the entire curated set (which should reflect the biological background distribution).

We selected a set of 177 RNA graphs containing up to 25 vertices from the curated data set (S1 Table), and created an expanded set by embedding subgraphs, randomly selected according to probability of occurrence, from the decoy database into these RNA graphs until each RNA graph contained 30 vertices. As a control, we also created a decoy set of graphs with 30 vertices, by random embedding of subgraphs from the decoy database only, *i*.*e*., graphs with no information from real biological structures except the frequency of occurrence of subgraphs in the known structures. Both the expanded and the decoy graph sets should be completely free of size effects since they all have exactly the same number of stems. The two sets were mixed and graphs compared using the Extended Fingerprint Jaccard Similarity. There is only a minor decrease in performance (S3 Table, Extended Fingerprint Jaccard Similarity: AUC = 0.840) when compared to the results obtained from the classification of the original dataset (Fig 5, Extended Fingerprint Jaccard Similarity: AUC = 0.952). As expected, the decoy set of graphs have AUC values close to 0.5, indicating that the decoy structures are random with respect to each other.

### Runtime analysis

Determination of whether a query RNA graph contains a subgraph isomorphic to a specific graph in the structural motif library, is an NP-complete problem [48]. The brute-force comparison requires comparing the query RNA graph with every graph in the library, and its computational complexity is *O*(*nm^m^*), where *n* is the number of graphs in the library (55,728), and *m* is the number of edges in the query graph. The subgraph random sampling algorithm can be parallelized by simultaneously running independent instances on multiple processors. The algorithm identifies the fingerprint of all 206 curated RNA graphs in a reasonable time, especially when it is run on multiple cores (S5 Fig). The average runtime for calculating the fingerprint of RNAs in each functional family is shown in S4 Table.

## Discussion

A great deal of work has focused on identifying similar RNAs based on the comparison of RNA secondary structures. This is readily accomplished using approaches such as tree edit distance [22, 61] or string related measures such as those used in RNAshapes [7]. Other approaches include the information of sequence alignment and folding of RNA sequences, for example, Saito *et al*. developed an algorithm that clusters RNAs by all possible sequence alignments, and all possible secondary structures computed from dynamic programming and partition function calculations [62–64]. This approach correctly discriminated short RNA sequences (around 100 bases) from different families. Unfortunately, secondary structures, and in particular minimum free-energy predicted structures based on dynamic programming approaches, do not predict pseudoknots [65–67], which are important in biological structures. Even if predicted pseudoknots are available [68–70], it is not simple to add them to tree or string based methods because of their non-nested nature. In addition, structure matching methods based on dynamic programming have the additional problem of determining gap penalties; it is not at all clear how to weight insertions and deletions in RNA structures.

Statistical algorithms, such as kernel methods, have been developed to classify RNA sequences and structures. Kin *et al*. developed a marginalized kernel to measure RNA sequence similarity [71], and this kernel was later implemented by Karklin *et al*. to measure the similarity of RNA secondary structures represented by dual graphs [29, 72]; Liu *et al*. developed a fuzzy kernel to cluster the secondary structure ensemble generated from a single sequence [73]. The GraphClust pipeline developed by Heyne *et al*. encodes RNA sequence-structure information as graphs and measures RNA graph similarities using a decomposition kernel and computing the summed similarity of pairs of neighborhood subgraphs [27]. However, no pseudoknotted structures were included in these approaches. Sakakibara *et al*. developed a stem kernel that could discriminate between functional RNA sequences and randomly shuffled sequences using structural features including pseudoknots [74]; however, no result was shown in which the stem kernel could discriminate between sequences from different functional RNA groups, in addition, the randomly shuffled sequences they generated only retain nucleotide composition, while preserving dinucleotide composition is known to be important in generating randomized negative controls for predicted RNA structures [75, 76]. In summary, none of these approaches have demonstrated that they can succeed on the difficult test case presented here: classifying a diverse set of functional families, with diverse sizes, containing pseudoknots, and with little sequence similarity. Topological methods have the dual advantage of easily representing pseudoknots and not requiring an insertion/deletion penalty. In the RNA-As-Graphs procedure [29, 31, 77, 78], RNA topologies are represented either as “tree graphs” (without pseudoknots) or “dual graphs” (with pseudoknots), and the topological properties of an RNA graph are summarized using the eigenvalue of its Laplacian matrix (constructed from the adjacency and degree matrices of the graph). They have developed a database, with all mathematically possible RNA graphs enumerated, including “existing graphs” (RNA structures experimentally solved or obtained from comparative analysis) and “missing graphs” (mathematically possible RNA structures that have not yet been experimentally observed). Using “existing graphs” as training data, “missing graphs” in the database were classified as either “RNA-like” or “non-RNA-like” by applying regression analysis on Laplacian eigenvalue spectra [34]. These approaches, which target the identification of novel RNA topologies, however, are not sufficient for matching specific RNA functional families.

Graph matching is a computationally intensive process that scales exponentially with the size of graph (in general, graph matching is an NP-hard process) [79]. Functional RNA molecules can include dozens of stems/loops, especially with the current advance in high-throughput technologies, and long non-coding RNAs including hundreds of stems/loops are not uncommon [80]. The RAG database, however, only includes dual graphs up to 9 vertices and tree graphs up to 10 vertices [77], which can cover RNA topologies only up to about 200nt, while the XIOS graph approach can handle RNA topologies with 70 vertices and 2000nt (S1 and S5 Tables). Moreover, in a follow-up study, the discrimination between structures predicted to be RNA-like (naturally existing) and non-RNA-like was not impressive; out of 42 newly discovered RNA topologies, only 24 of them had been predicted as RNA-like, while 18 of them had been predicted to be non-RNA like [77]. In addition, the numerical descriptors used in the RAG procedures have never been shown to be able to group RNAs into structural/functional classes.

The XIOS graph is a topological graph approach [36] that specifically distinguishes pseudoknots as a distinct type of edge. In addition to incorporating pseudoknots (O edge, Overlapping), one of the most important characteristics in RNA structure, the XIOS approach also includes embedding (I edge, Included) and juxtaposition (S edge, Serial), which are the two of the RNA structural principles in the RNAshapes framework. The increased number of edge-types in XIOS improves one’s ability to match graphs, for example using gSpan; however, the time required to find the maximal common subgraph in two moderately large RNA graphs, for instance with twenty to thirty stems in each graph, is prohibitive using exhaustive approaches such as gSpan. Using the XIOS approach, we can easily enumerate a complete set of biologically possible RNA graphs, permitting the construction of a complete dictionary of all graphs that may occur in a RNA molecule up to a specified size. This allows us to characterize any RNA topology in terms of the spectrum of subgraphs it contains, its RNA fingerprint, and to identify topologically similar RNA structures based on their fingerprints. This approach is successful with known RNA families, and is relatively insensitive to both the completeness of the RNA graph, and the presence of extraneous added vertices in the graph. Similarities between RNA structures in the same family are still detectable when the graphs are expanded to the same size, indicating that the ability to identify topologically similar structures is not simply an artifact of the similar sizes of RNAs within known families. These characteristics of RNA fingerprint matching are highly important in real-world settings where comparisons are made between predicted structures in which only 60–80% of the true stems may be correctly predicted [58, 81], and a substantial number of mispredicted stems may be present. As mentioned before, no previously reported RNA structure comparison method has shown that it can accurately identify/classify RNAs according to topological similarity using the particularly difficult set of pseudoknot containing graphs used here.

Exhaustively enumerating the set of subgraphs present in a XIOS graph is time consuming because each subgraph in the entire motif database must be separately tested against the query to determine whether there is a match. Because the dictionary of subgraphs is large (55,728 graphs with seven or fewer stems), a brute force approach is slow. In this work we suggest a sampling approach to enumerating the subgraph spectrum. The computational complexity of motif sampling depends on both the size and structure of the query graph, and on the number of vertices sampled in each iteration. As most of RNA XIOS graphs are highly connected, an increase in graph size can result in a large increase in the time required to completely sample the fingerprint. Fortunately, the motif sampling is completely parallelizable; any number of processors can independently sample subgraphs from the query, and the time required per query graph is modest. Furthermore, our results suggest that a complete fingerprint may not be necessary; that even incomplete fingerprints (such as fingerprints derived from structures where part of the structure has been removed) are sufficient to identify topologically similar structures. The question of whether absolutely every subgraph has been detected, which a sampling strategy cannot guarantee, is therefore somewhat moot.

Experimental determination of RNA structures by X-ray crystallography or NMR is difficult, and a relatively small number of complete structures are available. Instead, structures are often predicted using a combination of biochemical information (chemical modification, nuclease sensitivity, and mutational sensitivity), secondary structure prediction, and phylogenetic conservation (covariance). This results in “known” structures that are incomplete (missing important stems) or inaccurate (containing stems that do not exist, or are unimportant in the function of the RNA). It is therefore important that the structural/topological comparison be robust with respect to incompleteness or error in the structures, a salient characteristic of the RNA fingerprint comparison we describe here. The extended-fingerprint Jaccard Similarity correctly identifies topologically similar RNAs across a broad range of sizes, and biological functions, but its potential application is far more general. RNA structure prediction is commonly judged to be 60 to 80 percent accurate [59, 68, 82]. The ability of the RNA fingerprint to correctly identify/classify structural topologies even when 30% or more of the true stems are removed (Fig 5F), suggests that this approach can be applied to broadly search for topologically similar structures based on structures predicted from sequence (work in progress). Currently, GraphClust [27] is probably the most widely used program for comparing and clustering RNAs according to sequence and structure without pseudoknots. We believe that our approach, as a coarse-grained method including pseudoknots, complements that of GraphClust.

## Supporting Information

S1 Fig XIOS RNA graph representation of a Hepatitis D Virus (HDV) ribozyme RNA. (PDF)

S2 Fig Numbers of motifs in Simple and Extended Fingerprints. (PDF)

S3 Fig Heatmap dendrogram. (PDF)

S4 Fig Neighbor-joining tree showing the classification using Extended Jaccard Similarity. (PDF)

S5 Fig Runtime analysis of the subgraph random sampling algorithm. (PDF)

S1 Table Curated RNA structures. (PDF)

S2 Table Classification performance of Extended Fingerprint Jaccard Similarity for 8 curated families. (PDF)

S3 Table Classification performance for expanded graphs using different similarity functions. (PDF)

S4 Table Run time analysis. (PDF)

S5 Table Complete list of curated RNA structures used in this study. (XLSX)
