# Supplementary material for: Accurate Classification of RNA Structures Using Topological Fingerprints
Source: PLoS One. 2016 Oct 18;11(10):e0164726. doi: 10.1371/journal.pone.0164726 (PMC5068708; doi:10.1371/journal.pone.0164726)
Supplement: S1 Fig — (PDF) [file pone.0164726.s001.pdf]

S1 Fig. XIOS RNA graph representation of a Hepatitis D Virus (HDV) ribozyme RNA. The 3D and 2D structures are adapted from Ke et al [1]. (A) 3D structure; (B) 2D structure; (C) XIOS graph representation.

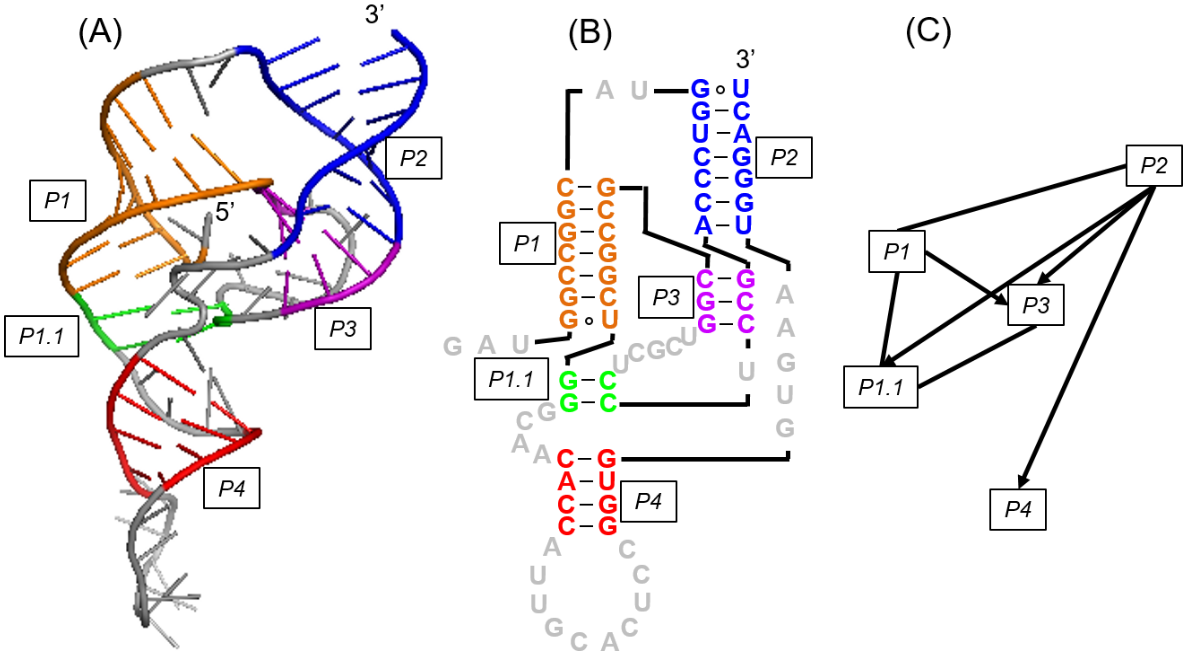

Reference

1. Ke A, Ding F, Batchelor JD, Doudna JA. Structural roles of monovalent cations in the HDV ribozyme. Structure. 2007;15(3):281-7. doi: 10.1016/j.str.2007.01.017. PubMed PMID: 17355864.
