# Supplementary material for: Accurate Classification of RNA Structures Using Topological Fingerprints
Source: PLoS One. 2016 Oct 18;11(10):e0164726. doi: 10.1371/journal.pone.0164726 (PMC5068708; doi:10.1371/journal.pone.0164726)

S2 Fig. Numbers of motifs in Simple and Extended Fingerprints. The average number of motifs in the Simple Fingerprint (upper pink bars, determined by subgraph sampling) or the Extended Fingerprint (lower blue bars, determined by combining sampled subgraphs and all the ancestral subgraphs cataloged in the motif library) in different RNA families a shown beside the corresponding bar. Error bars show the standard deviation of number of motifs.

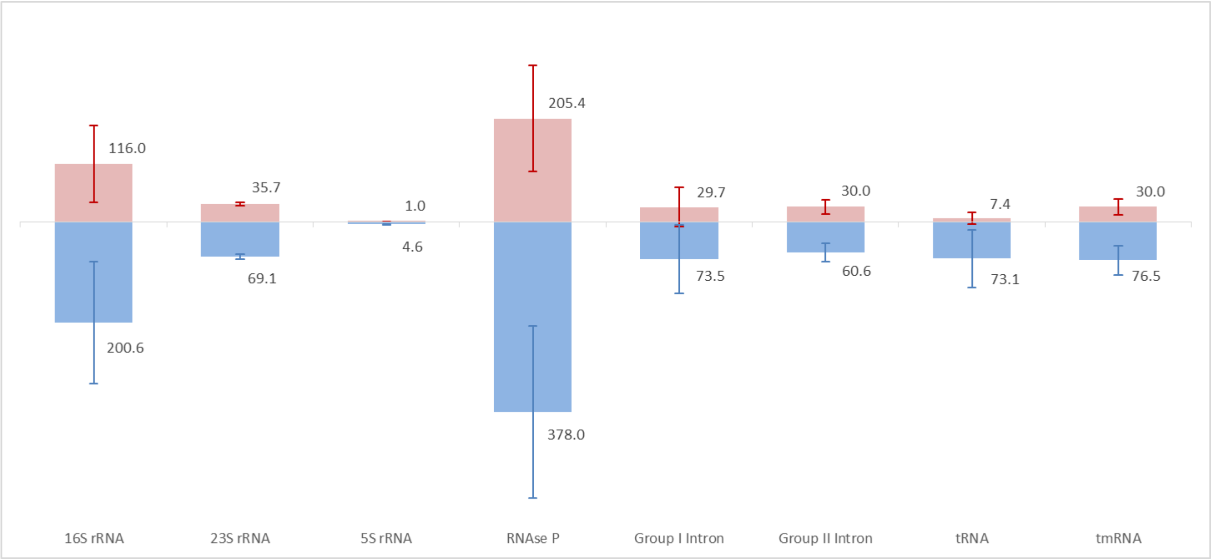

Supplement: S2 Fig — (PDF) [file pone.0164726.s002.pdf]
