# Supplementary material for: Accurate Classification of RNA Structures Using Topological Fingerprints
Source: PLoS One. 2016 Oct 18;11(10):e0164726. doi: 10.1371/journal.pone.0164726 (PMC5068708; doi:10.1371/journal.pone.0164726)
Supplement: S3 Fig — (PDF) [file pone.0164726.s003.pdf]

S3 Fig. Heat map dendrogram. (Same as Fig 6, but with RNA names shown) This figure shows the heat map dendrogram of sequence similarity (upper-left triangle) and fingerprint similarity (Extended Jaccard Similarity, lower-right triangle) of all the curated RNA structures (represented by IDs followed by their names, corresponding to S5 Table). Similarity is shown in different colors, ranging from 0 (blue) to 1 (red) at steps of 0.1. A neighbor-joining tree calculated according to the fingerprint similarity is shown on the right side of the heat map (branching showing the tree topology but branch lengths are not the true distances between nodes).

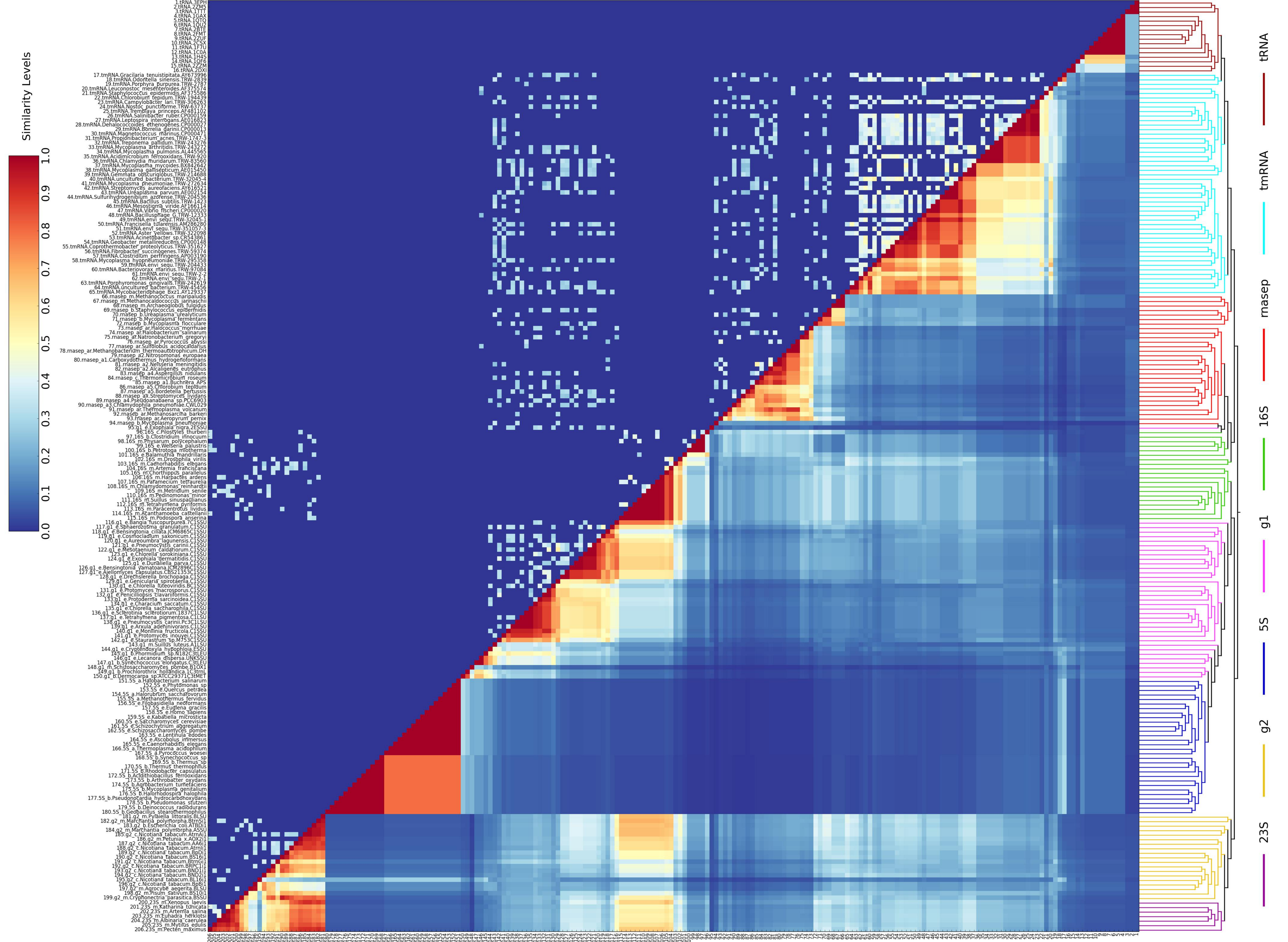
Similarity Levels
1.0
0.9
0.8
0.7
0.6
0.5
0.4
0.3
0.2
0.1
0.0
tRNA
tmRNA
rnasep
16S
g1
5S
g2
23S
