# Supplementary material for: Accurate Classification of RNA Structures Using Topological Fingerprints
Source: PLoS One. 2016 Oct 18;11(10):e0164726. doi: 10.1371/journal.pone.0164726 (PMC5068708; doi:10.1371/journal.pone.0164726)

**S4 Fig. Neighbor-joining tree showing the classification using Extended Jaccard Similarity.** See S3 Fig and S5 Table for IDs. The labeled branch is a misclassified RNA: Group I Introns from Exophiala nigra (eukaryotic nucleus) (94).

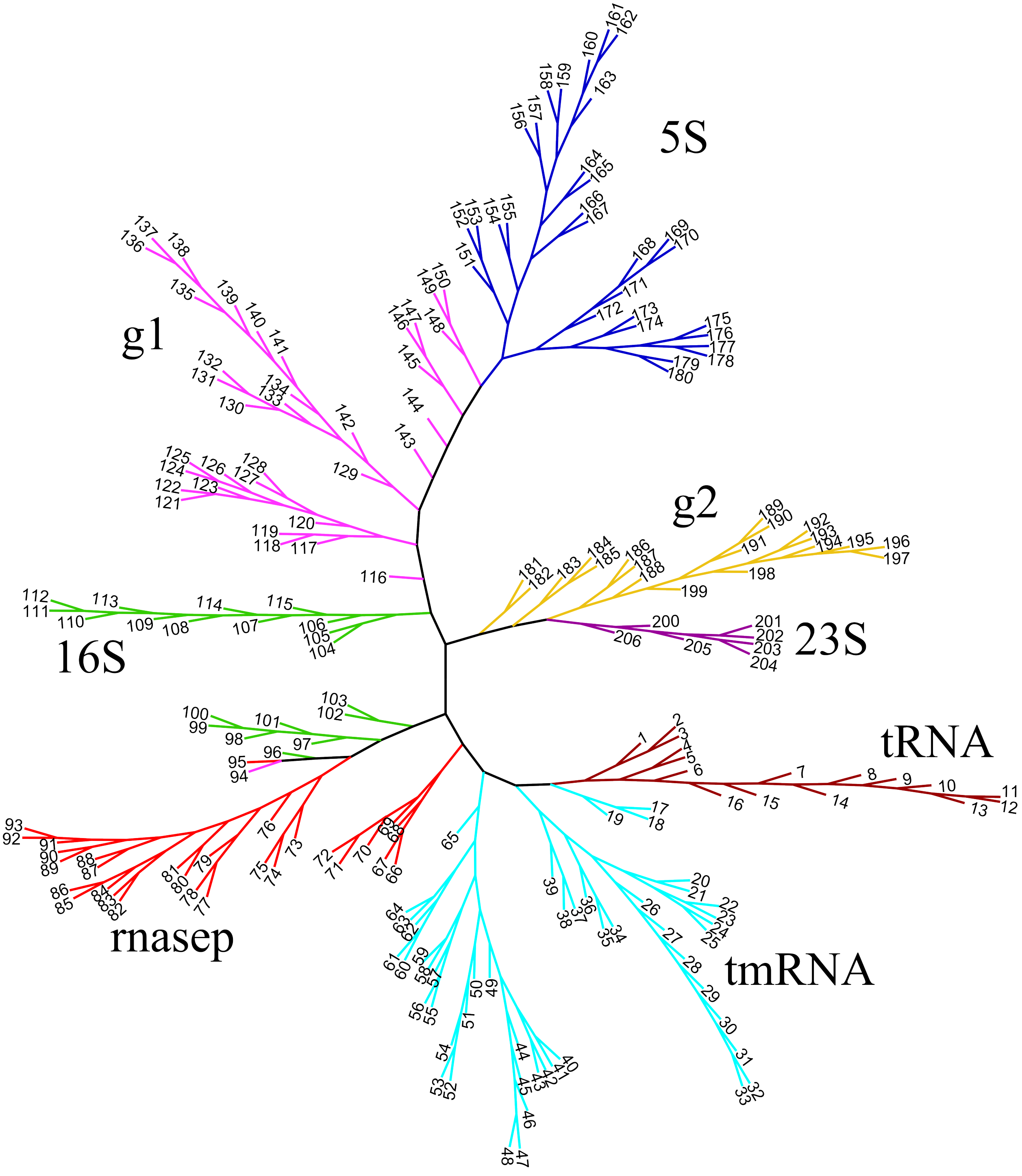

Supplement: S4 Fig — (PDF) [file pone.0164726.s004.pdf]
