# Supplementary figures and images for: Accurate Classification of RNA Structures Using Topological Fingerprints
Source: PLoS One. 2016 Oct 18;11(10):e0164726. doi: 10.1371/journal.pone.0164726 (PMC5068708; doi:10.1371/journal.pone.0164726)

**S5 Fig. Runtime analysis of the subgraph random sampling algorithm.**

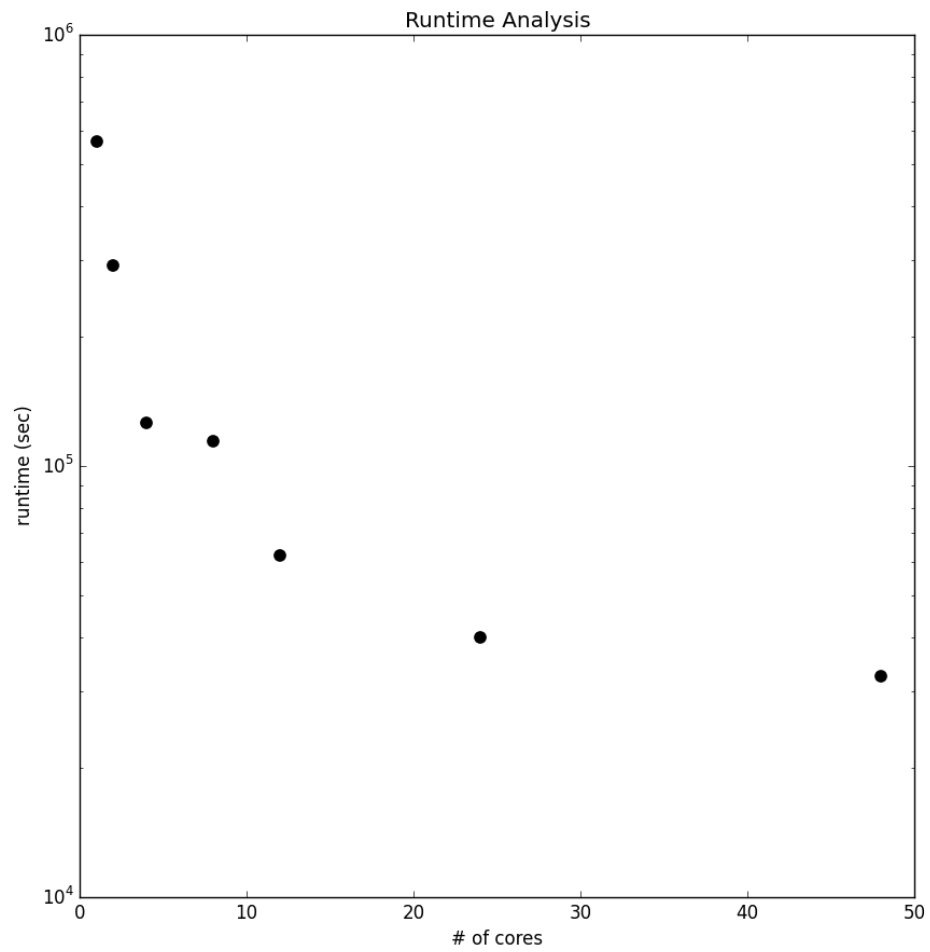

Supplement: S5 Fig — (PDF) [file pone.0164726.s005.pdf]
