# Supplementary material for: Accurate Classification of RNA Structures Using Topological Fingerprints
Source: PLoS One. 2016 Oct 18;11(10):e0164726. doi: 10.1371/journal.pone.0164726 (PMC5068708; doi:10.1371/journal.pone.0164726)
Supplement: S1 Table — (PDF) [file pone.0164726.s006.pdf]

S1 Table. Curated RNA structures.

RNA Family	N	Vertices	Edges	Average Degree	Description
tRNA	16	7.5(0.9)	15.2(3.9)	4.0(0.5)	Transfer RNAs with resolution < 3 Å from the Protein Data Bank (PDB) [1, 2]. Base pairing information calculated with RNAView [3]. The following PDB IDs are included: 1C0A, 1F7U, 1GAX, 1H4S, 1QF6, 1QTQ, 1QU2, 1TTT, 2BTE, 2CSX, 2DXI, 2FMT, 2ZM5, 2ZUF, 2ZZM, 3EPH.
RNase P RNA	29	16.7(3.1)	72.8(26.0)	8.5(1.7)	Representative Ribonuclease P RNA structures from the classes enumerated by Ellis and Brown [4]. Secondary structures and pseudoknots were assigned according to Ellis and Brown [5].
tmRNA	49	14.0(2.5)	44.3(14.4)	6.2(1.2)	Transfer-messenger (10Sa) RNA. Aligned tmRNA sequences and structural assignments were obtained from Mao et al [6].
Group I Intron RNA	36	17.4(3.6)	26.2(9.4)	2.9(0.7)	Group I Self-Splicing Intron RNA. Sequences and structural assignments were obtained from the Comparative RNA Web site [7]; the shortest and longest 10% in length were removed to avoid incomplete or poorly annotated sequences. Containing 3 subgroups: b (bacteria), e (eukaryotic nucleus), and m (eukaryotic mitochondria).
Group II Intron RNA	19	21.0(4.8)	42.9(17.7)	4.0(0.7)	Group II Self-Splicing Intron RNA. Sequences and structural assignments were from the Comparative RNA Web site [7]. Containing 3 subgroups: b (bacteria), c (eukaryotic chloroplast), and m (eukaryotic mitochondria).
5S rRNA	30	4.6(0.5)	5.1(1.0)	2.2(0.2)	5S Ribosomal RNA sequences and structural assignments were obtained from CRW Site [7]. Containing 3 subgroups: a (archaea), b (bacteria), e (eukaryotic nucleus).
16S rRNA	20	51.9(14.7)	171.6(74.0)	6.3(1.2)	16S Ribosomal RNA sequences and structural assignments were obtained from CRW Site [7]. Containing 4 subgroups: b (bacteria), c (eukaryotic chloroplast), e (eukaryotic nucleus), and m (eukaryotic mitochondria).
23S rRNA	7	50.4(8.4)	95.0(19.2)	3.7(0.1)	23S Ribosomal RNA sequences and structural assignments were obtained from CRW Site [7]. Containing 1 group: m (eukaryotic mitochondria).

Curated RNA structures with graph characteristics (vertex number, edge number, average degree, and curation description).

References

1. Berman HM, Westbrook J, Feng Z, Gilliland G, Bhat TN, Weissig H, et al. The Protein Data Bank. Nucleic Acids Res. 2000;28(1):235-42. PubMed PMID: 10592235; PubMed Central PMCID: PMCPMC102472.
2. Rose PW, Beran B, Bi C, Bluhm WF, Dimitropoulos D, Goodsell DS, et al. The RCSB Protein Data Bank: redesigned web site and web services. Nucleic Acids Research. 2011;39:D392-D401. doi: 10.1093/nar/gkq1021. PubMed PMID: WOS:000285831700065.
3. Yang HW, Jossinet F, Leontis N, Chen L, Westbrook J, Berman H, et al. Tools for the automatic identification and classification of RNA base pairs. Nucleic Acids Research. 2003;31(13):3450-60. doi: 10.1093/nar/gkg529. PubMed PMID: WOS:000183832900036.
4. Ellis JC, Brown JW. The RNase P family. Rna Biology. 2009;6(4):362-9. PubMed PMID: WOS:000275572500002.
5. Brown JW. The Ribonuclease P Database. Nucleic Acids Research. 1999;27(1):314-. doi: 10.1093/nar/27.1.314. PubMed PMID: WOS:000077983000085.
6. Mao C, Bhardwaj K, Sharkady SM, Fish RI, Driscoll T, Wower J, et al. Variations on the tmRNA gene. RNA Biol. 2009;6(4):355-61. PubMed PMID: 19617710.
7. Cannone JJ, Subramanian S, Schnare MN, Collett JR, D'Souza LM, Du YS, et al. The Comparative RNA Web (CRW) Site: an online database of comparative sequence and structure information for ribosomal, intron, and other RNAs. Bmc Bioinformatics. 2002;3:31. doi: 210.1186/1471-2105-3-2. PubMed PMID: WOS:000181476800002.
