# Supplementary material for: Accurate Classification of RNA Structures Using Topological Fingerprints
Source: PLoS One. 2016 Oct 18;11(10):e0164726. doi: 10.1371/journal.pone.0164726 (PMC5068708; doi:10.1371/journal.pone.0164726)
Supplement: S2 Table — (PDF) [file pone.0164726.s007.pdf]

S2 Table. Classification performance of Extended Fingerprint Jaccard Similarity for 8 curated families.

RNA Family	Area Under Curve (AUC)
5S rRNA	1.000
16S rRNA	0.915
23S rRNA	0.996
RNase P	0.957
group I Intron	0.898
group II Intron	0.952
tRNA	1.000
tmRNA	0.974
total	0.952
