# Supplementary material for: Accurate Classification of RNA Structures Using Topological Fingerprints
Source: PLoS One. 2016 Oct 18;11(10):e0164726. doi: 10.1371/journal.pone.0164726 (PMC5068708; doi:10.1371/journal.pone.0164726)
Supplement: S3 Table — (PDF) [file pone.0164726.s008.pdf]

S3 Table. Classification performance for expanded graphs using different similarity functions.

Similarity Function	SimFP total (decoy only)	ExtFP total (decoy only)
Intersection Similarity	0.699 (0.501)	0.694 (0.481)
Cosine Similarity	0.666 (0.524)	0.654 (0.560)
Dice Similarity	0.806 (0.517)	0.827 (0.500)
Hamming Similarity	0.698 (0.542)	0.721 (0.563)
Jaccard Similarity	0.794 (0.515)	0.840 (0.524)

The value outside of parentheses is area under curve (AUC) for expanded graphs from curated structures; the value inside of parentheses is AUC for decoy graphs, which are considered random and used as controls.
