# Supplementary material for: Accurate Classification of RNA Structures Using Topological Fingerprints
Source: PLoS One. 2016 Oct 18;11(10):e0164726. doi: 10.1371/journal.pone.0164726 (PMC5068708; doi:10.1371/journal.pone.0164726)
Supplement: S4 Table — (PDF) [file pone.0164726.s009.pdf]

**S4 Table. Run time analysis.**

| Family \ # of cores | 1 | 2 | 4 | 8 | 12 | 24 | 48 |
|---|---|---|---|---|---|---|---|
| **16S** | 59289 | 30113 | 14916 | 11563 | 5598 | 4267 | 2976 |
| **23S** | 3638 | 2533 | 1088 | 465 | 389 | 192 | 211 |
| **5S** | 0 | 0 | 0 | 0 | 0 | 0 | 1 |
| **g1** | 3323 | 1837 | 880 | 549 | 327 | 232 | 194 |
| **g2** | 3265 | 1728 | 884 | 509 | 300 | 219 | 196 |
| **rnasep** | 190229 | 97012 | 45339 | 32373 | 18631 | 14114 | 10434 |
| **tRNA** | 1165 | 540 | 269 | 175 | 101 | 75 | 52 |
| **tmRNA** | 3627 | 1822 | 967 | 662 | 331 | 221 | 204 |

This table shows the average runtime (unit: seconds, rounded to the nearest integer) versus different number of cores for the subgraph sampling algorithm to calculate the fingerprints in each functional RNA family. The runtime reduces as the number of cores increases.
